# Gas-Sensing Performance of M-Doped CuO-Based Thin Films Working at Different Temperatures upon Exposure to Propane

**DOI:** 10.3390/s150820069

**Published:** 2015-08-14

**Authors:** Artur Rydosz, Aleksandra Szkudlarek

**Affiliations:** 1 AGH University of Science and Technology, Av. Mickiewicza 30, 30-059 Krakow, Poland; 2 Academic Centre for Materials and Nanotechnology, AGH University of Science and Technology, Av. Mickiewicza 30, 30-059 Krakow, Poland; E-Mail: aleksandra.szkudlarek@agh.edu.pl

**Keywords:** gas sensor applications, CuO films, magnetron sputtering, metal doping thin films, propane

## Abstract

Cupric oxide (CuO) thin films are promising materials in gas sensor applications. The CuO-based gas sensors behaved as p-type semiconductors and can be used as part of an e-nose or smart sensor array for breath analysis. The authors present the investigation results on M-doped CuO-based (M = Ag, Au, Cr, Pd, Pt, Sb, Si) sensors working at various temperatures upon exposure to a low concentration of C_3_H_8_, which can be found in exhaled human breath, and it can be considered as a one of the biomarkers of several diseases. The films have been deposited in magnetron sputtering technology on low temperature cofired ceramics substrates. The results of the gas sensors’ response are also presented and discussed. The Cr:CuO-based structure, annealed at 400 °C for 4 h in air, showed the highest sensor response, of the order of 2.7 at an operation temperature of 250 °C. The response and recovery time(s) were 10 s and 24 s, respectively. The results show that the addition of M-dopants in the cupric oxide films effectively act as catalysts in propane sensors and improve the gas sensing properties. The films’ phase composition, microstructure and surface topography have been assessed by the X-ray diffraction (XRD), scanning electron microscopy (SEM) and energy dispersive X-ray spectroscopy (EDX) methods.

## 1. Introduction

Exhaled human breath is a complex mixture of inorganic gases (e.g., NO, CO_2_ and CO), volatile organic compounds (VOCs) (e.g., isoprene, acetone, propane) and other typically non-organic volatile substances (e.g., N_2_) [1]. VOCs are mainly in the parts per million (ppm) or parts per billion (ppb) range. The composition of exhaled breath gas depends on numerous variables, like life style, nutrition, activity, inhaled air composition, *etc.* Some of the VOCs are named “biomarkers”, since their presence in breath indicates disease. Breath analysis has many advantages over conventional laboratory tests. It is non-invasive and can be repeated frequently without any risk to the patient. The total number of diseases that can be detected by breath analysis is still unknown. Numerous studies, which appeared in the last few decades, correlate the presence of VOCs in breath to a certain disease, *i.e.*, asthma [2], COPD (chronic obstructive pulmonary disease) [3], lung cancer [4], metabolic disorder [5], oxidative stress and others [6]. One of the VOCs that can be found in exhaled human breath is a propane C_3_H_8_ (Chemical Abstract Service: 74-98-6). Mostly, it is exhaled by a patient with oxidative stress [7] and lipid peroxidation of unsaturated fatty acids [8]. Barker *et al.* [9] have reported investigation results on trace analysis from 20 cystic fibrosis patients and 20 healthy controls. All subjects were nonsmokers. The mean value of exhaled propane was 1.95 ppb for both groups. Kulikov *et al.* [10] have presented investigation results on trace analysis of light hydrocarbons (C_2_-C_3_) from patients with type 2 diabetes mellitus (T2DM). They concluded that light hydrocarbons are intermediate or side products of many metabolic cycles, and therefore, they may be an indicator of metabolism disorder, e.g., T2DM. Kischkel *et al.* [11] have reported investigation results on breath profiles from 31 lung cancer patients, 31 smokers and 31 healthy controls. The exhaled propane was in the 0.35–10.09 nmoL/L range. However, the exhaled concentrations did not show any statistical significances between the study groups. Rudnicka *et al.* [12] have reported investigation results on biomarkers of lung cancer detected in human breath of smoking and non-smoking volunteers and patients with diagnosed lung cancer. The total number of persons in which propane was identified was nine and 24 for non-smokers and smokers, respectively. The concentration range of propane was 3.45 ppb–5.96 ppb and 3.19 ppb–9.74 ppb for healthy persons and lung cancer patients, respectively. The results indicated that the level of propane can be higher for patients with a lung cancer with respect to healthy volunteers, especially for smokers. Recently, Bujak *et al.* [13] have reported research results on biomarkers of carious organism disorders. The propane was recognized as a biomarker of asthma for children [13]. Therefore, the propane sensors can be used as part of an e-nose or smart sensor array for breath analysis. However, commercially available propane sensors are developed for measuring samples at several tens of parts per million (ppm). Hence, many researchers have focused on the investigation on semiconductive oxides with higher sensitivity to propane, *i.e.*, SnO_2_ [14], ZnO [15], CeO_2_ [16], *etc.* Aguilar-Leyva *et al.* [17] have presented the gas-sensitive properties of SnO_2_ thin films, as well as Ag/SnO_2_ and SnO_2_/Ag structures in an atmosphere containing propane. The sensors were measured in a propane atmosphere with different gas concentrations, *i.e.*, 50–500 ppm. The best results were obtained for Ag/SnO_2_ structures. The sensitivity is defined as: *S = (R*_g_*−R*_0_*)/R*_0_, where *R*_0_ is the resistance of the sensor in the presence of air and *R*_g_ is the resistance in the presence of propane gas. The obtained sensitivity was in the range of 300–400 for a wide range of propane concentrations (100–500 ppm). Castro *et al.* [18] have presented results on propane sensing obtained with 5 mol% Cd-doped SnO_2_. The tested concentrations were 0, 25, 50, 75 and 100 ppm, and *ΔR/R*_0_ increased regularly by approximately 0.02 at each 25 ppm concentration step. The sensitivity under exposure to 1 ppm and 100 ppm of propane is approximately 0.08 and 0.16, respectively. Saberi *et al.* [19] have reported results on the dual selective Pt/SnO_2_ sensor to propane. However, the authors have focused on an automotive application due to their measured sensors being under higher propane concentrations (1000–10,000 ppm). Even though, the maximum sensor response *R*_a_*/R*_g_ (where *R*_a_ is the resistance of the sensor in the presence of air and *R*_g_ is the resistance in the presence of propane gas) was approximately five. Sun *et al.* [20] have reported results on a Zn-M-O (M = Sn, Co) sensing electrode for selective propane sensors. The highest sensitivity (defined as mV/decade) was obtained for a Zn-Sn-O composited sensing electrode with 50% Pt coverage, and it was approximately 17. Liu *et al.* [21] have presented investigation results on a Pt-CeO_2_ nanofiber-based high-frequency impedancemetric gas sensor. The sensor response defined as log(*Z*_g_*/Z*_0_), where *Z*_0_ is the impedance of the sensor in the presence of air and *Z*_g_ is the impedance in the presence of propane gas, was approximately one under 20 ppm C_3_H_6_. However, it is still too high of a range in comparison to exhaled propane levels.

In this paper, novel M-doped CuO-based (M = Ag, Au, Cr, Pd, Pt, Sb, Si) sensors with enhanced sensitivity to propane have been presented. The long-term stability is described and discussed. The films’ phase composition, microstructure and surface topography have been assessed by X-ray diffraction (XRD), scanning electron microscopy (SEM) and energy dispersive X-ray spectroscopy (EDX) methods.

## 2. Experimental Section

### 2.1. Preparation of Films

**Figure 1:**
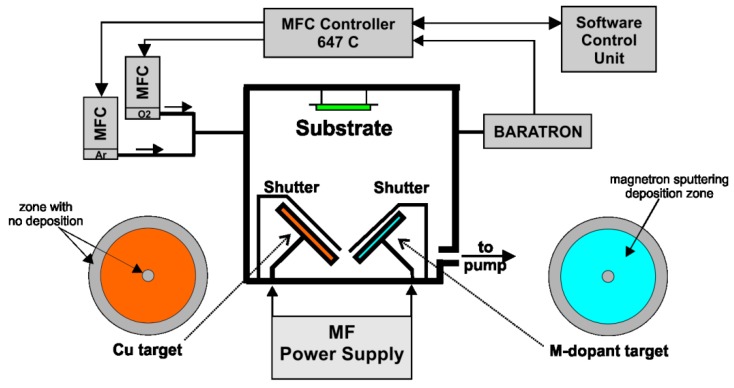
Schematic view of the medium frequency (MF) magnetron co-sputtering system with 50-mm magnetron targets.

**Figure 2:**
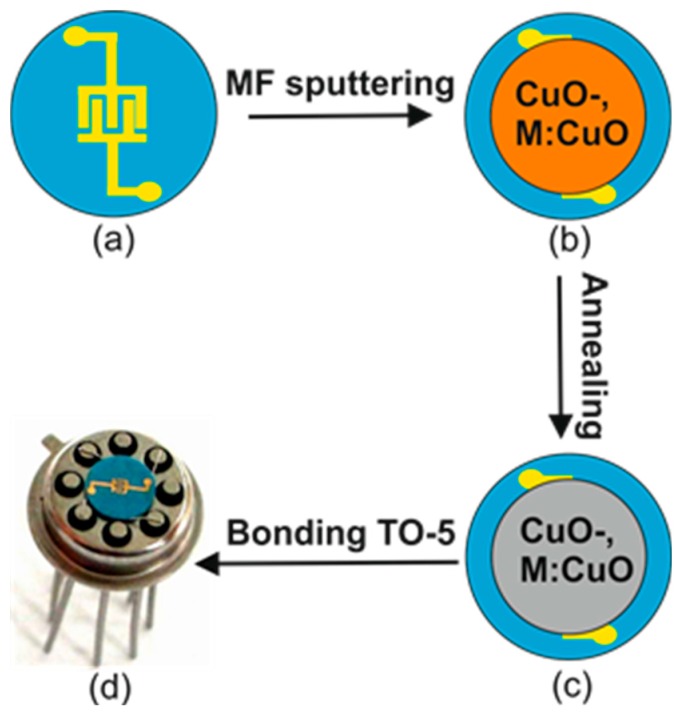
Schematic view of fabrication of gas sensors: (**a**) sensor substrate fabricated in low temperature cofired ceramic (LTCC) technology with interdigitated Au electrodes on the top; (**b**) CuO- and M:CuO-based thin films, obtained by MF sputtering deposition (the deposition parameters are in the text); (**c**) CuO- and M:CuO-based sensors after annealing (400 °C/4 h in air); (**d**) sensor mounted to the TO-5 package.

The reported films were deposited on silicon and LTCC (low temperature cofired ceramic) substrates, previously reported in [22] by an MF (medium frequency) magnetron co-sputtering system, schematically shown in Figure 1. The metallic copper target (purity 99.995%) and metallic dopants targets (99.95%) of 50 mm and 10 mm in diameter were used, respectively. The pressure was set to 2.0 × 10^−6^ mbar and 4.0 × 10^−2^ mbar for the base vacuum and the working pressure, respectively. The target to substrate distance was set to 50 mm. After the standard cleaning process, the gas-sensitive layers were deposited at 100 °C and then annealed at 500 °C for 4 h in air. The deposition and annealing step parameters were chosen based on previous results. The substrate temperature was controlled by PID Eurotherm 2408. The targets were pre-sputtered for 10 min to eliminate target surface contamination and to obtain a stable plasma density. Sputtering was then performed under both pure argon (Ar) and argon/oxygen (90% Ar/10% O_2_). The sputtering was completed with a low power of 40 W. The sputtering time was changed to yield different film thicknesses. However, the highest gas responses were obtained for a 50-nm film thickness. The responses of the other sensors were around 20%–50% lower than observed in the case of the sensor with an optimal thickness of 50 nm. The sputtering parameters were chosen based on previously-reported results [23] and controlled by homemade software with MFCs (mass flow controllers) and a Baratron pressure gauge (MKS Instruments^®^, Andover, MA, USA). Figure 2 shows the schematic view of preparing the gas sensors. Furthermore, the deposition parameters, such as pre-sputtering time, deposition temperature, power, *etc.*, were the same for CuO- and M:CuO-based thin films.

### 2.2. Film Characterization

The structural analysis of the films was carried out by an X-ray diffraction technique using PANalytical X’Pert Pro MDP with CuKα (λ = 1.5406 Å) at a step size of 0.04° over the 2Ɵ range of 30–80°. The chemical composition of the films was confirmed by energy dispersive X-ray (EDX) analysis using the FEI VERSA 3D system. The microstructure of the samples was characterized by scanning electron microscopy FEI VERSA 3D.

### 2.3. Gas Sensing

The gas-sensing performance of the deposited and annealed thin films for propane was examined using a homemade computer-controlled system. The gas concentrations were controlled by changing the mixing ratio of dry air and propane using mass flow controllers (MKS Instruments). The total flow rate was set to 500 sccm. The gas sensing response (*R*) was defined as *R = R*_gas_*/R*_air_, where *R*_gas_ and *R*_air_ are the electrical resistances in the presence and absence of propane, respectively.

## 3. Results and Discussion

### 3.1. X-Ray Diffraction

**Figure 3:**
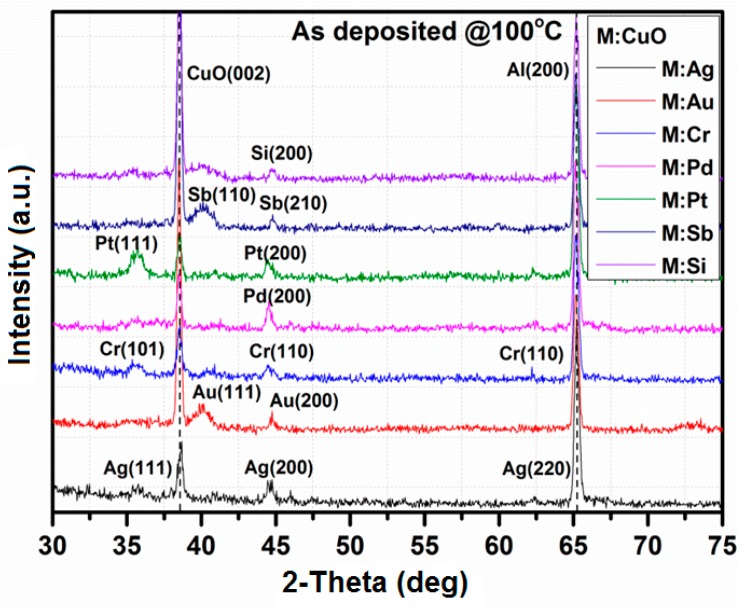
XRD patterns of M:CuO (M: Ag, Au, Cr, Pd, Pt, Sb, Si) doping agents after deposition at 100 °C.

**Figure 4:**
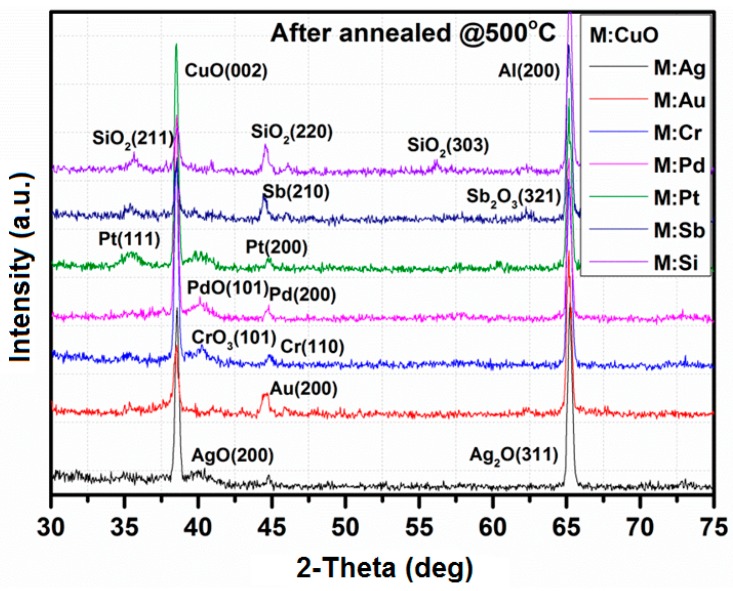
XRD patterns of M:CuO (M: Ag, Au, Cr, Pd, Pt, Sb, Si) doping agents after annealing at 500 °C for 4 h in air.

Figure 3 shows the XRD patterns of the films after deposition. All of the diffraction peaks were well indexed to the references. Table 1 shows crystallographic parameters for films after the annealing process. As can be notice, the films exhibit mostly a monoclinic or cubic crystallographic system. The silicon substrates used for material characterizations were previously coated by thick Al film (1000 nm) to avoid peaks from silicon for the Si:CuO-based sensor. Due to the characteristics, peaks from Al (200) can be observed. Figure 4 shows the XRD patterns of the films after annealing at 500 °C for 4 h in air. As seen from Figure 3, the XRD patterns obtained after annealing were crystallized with the presence of AgO (200), Ag_2_O (311), Au (200), CrO_3_ (101), Cr (110), PdO (110), Pd (200), Pt (111), Pt (200), Sb (210), Sb_2_O_3_ (321), SiO_2_ (211), SiO_2_ (220) and SiO_2_ (303) reflections for Ag-, Au-, Cr-, Pd-, Pt-, Sb- and Si-doped CuO, respectively. The presence of cooper and metal dopants was additionally confirmed by energy dispersive X-ray spectroscopy (EDX).

**Table 1:** Crystallographic parameters of thin films after annealing at 500 °C for 4 h in air.

Thin Film	Crystallographic Parameters						
	Crystal System	a (Å)	b (Å)	c (Å)	α (°)	β (°)	γ (°)
CuO	Monoclinic	4.6797	3.4314	5.1362	90	99.26	90
Au:CuO	Cubic	4.0786	4.0786	4.0786	90	90	90
Ag:CuO	Monoclinic	5.8500	3.4800	5.5000	90	107.5	90
Cr:CuO	Cubic	4.0400	4.400	4.0400	90	90	90
Pd:CuO	Cubic	3.8902	3.8902	3.8902	90	90	90
Pt:CuO	Hexagonal	3.1000	3.1000	8.3200	90	90	120
Sb:CuO	Cubic	6.1347	6.1347	6.1347	90	90	90
Si:CuO	Monoclinic	8.8664	4.7482	8.7918	90	115	90

### 3.2. SEM/EDX

**Figure 5:**
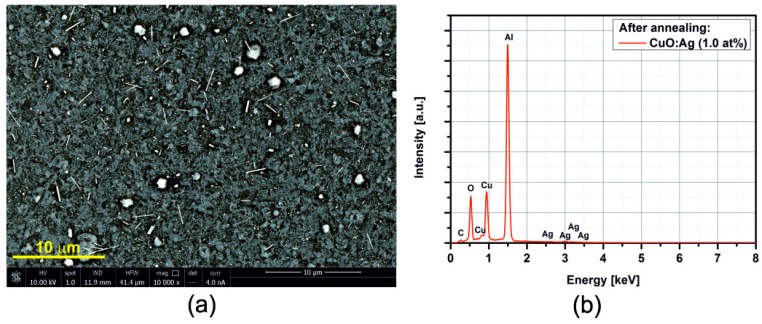
(**a**) SEM image; (**b**) EDX distribution of the Ag:CuO-based sensor (Ag: 1.0at%).

**Figure 6:**
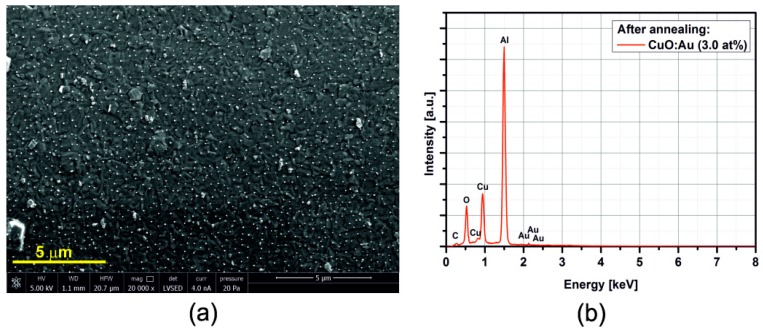
(**a**) SEM image; (**b**) EDX distribution of the Au:CuO-based sensor (Au: 3.0at%).

**Figure 7:**
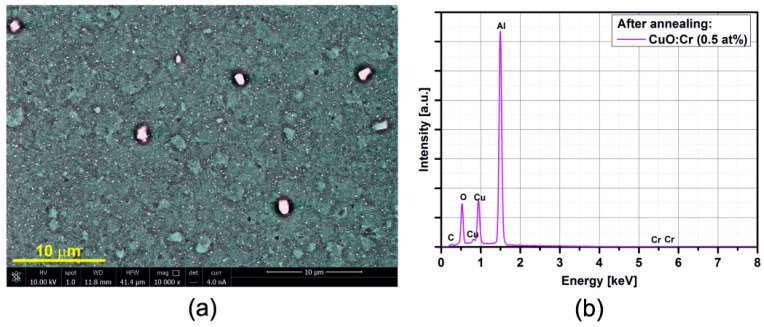
(**a**) SEM image; (**b**) EDX distribution of the Cr:CuO-based sensor (Cr: 0.5at%).

**Figure 8:**
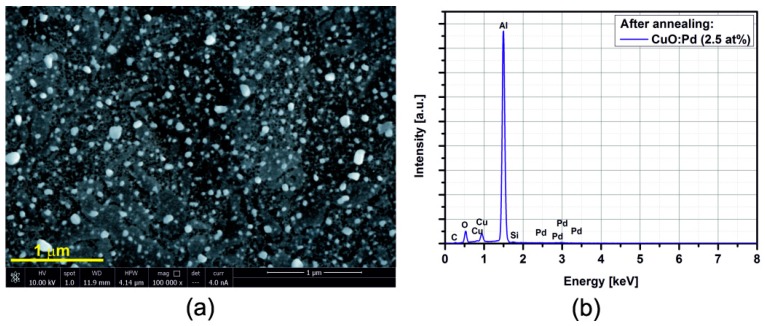
(**a**) SEM image; (**b**) EDX distribution of the Pd:CuO-based sensor (Pd: 2.5at%).

**Figure 9:**
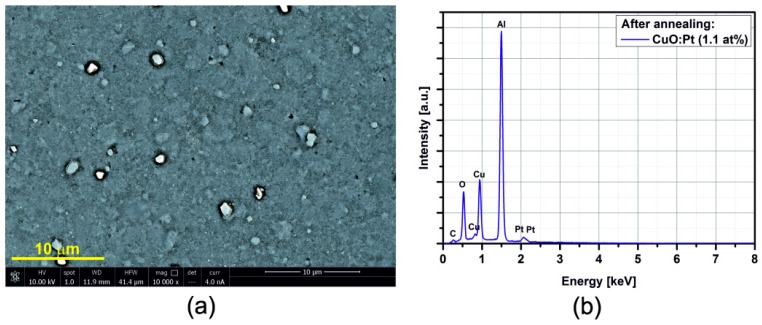
(**a**) SEM image; (**b**) EDX distribution of the Pt:CuO-based sensor (Pt: 1.1at%).

**Figure 10:**
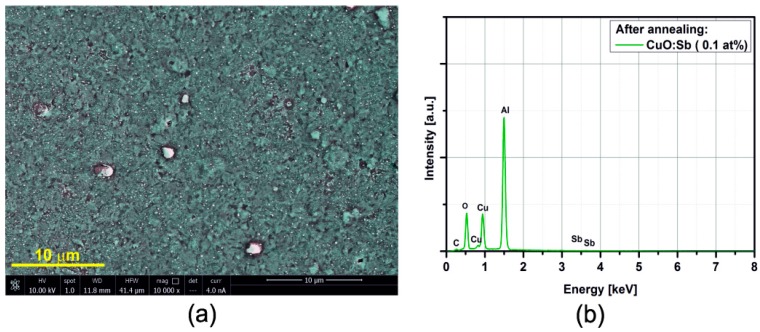
(**a**) SEM image; (**b**) EDX distribution of the Sb:CuO-based sensor (Sb: at%).

**Figure 11:**
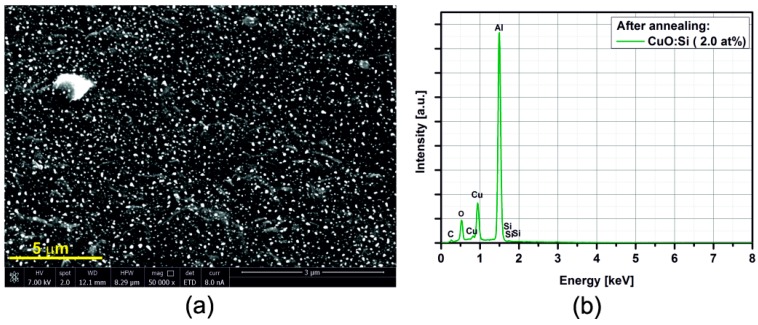
(**a**) SEM image; (**b**) EDX distribution of the Si:CuO-based sensor (Si: 2.0 at%).

Figure 5, Figure 6, Figure 7, Figure 8, Figure 9, Figure 10 and Figure 11 show the SEM images (a) and EDX distribution (b) of the M:CuO-based sensor (M = Ag, Au, Cr, Pd, Pt, Sb, Si). The EDX results display the presence of carbon, oxygen, copper and dopant elements in the analyzed sample. No significant change in the relative content of the four elements suggests a stable elemental distribution in the M:CuO-based sensors. A low-magnification SEM image (Figure 5a) demonstrates that the Ag:CuO-based nanostructure consists of heterogeneous particles with various sizes of about 0.5–3 μm. As can be noticed in Figure 6a, the gold dopants are uniform spheres with a diameter around 75 nm and are uniformly distributed over the Au:CuO-based sensor. The same results were obtained for Pd:CuO-based (Figure 8a) and Si:CuO-based (Figure 11a) sensors. However, the Cr:CuO-based (Figure 7a), Pt:CuO-based (Figure 9a) and Sb:CuO-based (Figure 10a) sensors exhibit different dopant crystal dimensions, shapes and distribution. They are in the range of several nm to several μm, which can be related to the low substrate temperature during the deposition.

### 3.3. Electrical Resistance Measurements

It is well known that electrical resistance decreases when temperature increases for semiconductor gas sensors. The basic approaches for metal oxide gas sensors are widely presented in the literature, e.g., [24,25]. In this study, the electrical resistance of the samples was measured in the amperometric mode by using the Keithley 6517 electrometer. Table 2 shows the baseline resistances in air (RH: 50%) at various temperatures for CuO- and M:CuO-based sensors. The resistance can be written as: R(T) = R_0_[1 + α (T − T_0_)], where R_0_ is the resistance at temperature T_0_ and α is the resistance temperature coefficient. The calculated temperature coefficient of resistance (TCR) was in the range of 0.0045–0.4400 (1/°C). However, the TCRs of M:CuO films were at least ten times higher than for pure CuO. Cheng *et al.* [26] have reported the TCR of single-crystalline CuO nanowires to be approximately 0.0075 (1/°C).

**Table 2:** The baseline resistances in air at various temperatures for 50-nm thin films deposited at 100 °C and annealed at 500 °C for 4 h in dry air.

Thin Film	Baseline Resistance at Temperature (°C)				Temperature Coefficient of Resistance (1/°C)
	180	250	320	380	
CuO	100 kΩ ± 0.5 kΩ	30 kΩ ± 0.15 kΩ	15 kΩ ± 0.07 Ω	9 kΩ ± 0.04 Ω	−0.0045
Au:CuO	9000 kΩ ± 45 kΩ	1335 kΩ ± 7 kΩ	261 kΩ ± 1 kΩ	103 kΩ ± 0.5 kΩ	−0.4400
Ag:CuO	1060 kΩ ± 4 kΩ	405 kΩ ± 2 kΩ	140 kΩ ± 0.7 kΩ	80 kΩ ± 0.4 kΩ	−0.0490
Cr:CuO	530 kΩ ± 2.7 kΩ	140 kΩ ± 0.7 kΩ	45 kΩ ± 0.22 kΩ	30 kΩ ± 0.15 kΩ	−0.0250
Pd:CuO	103 kΩ ± 0.5 kΩ	45 kΩ ± 0.2 kΩ	18 kΩ ± 0.1 kΩ	11 kΩ ± 0.05 Ω	−0.0460
Pt:CuO	303 kΩ ± 1.5 kΩ	107 kΩ ± 0.6 kΩ	40 kΩ ± 0.2 kΩ	23 kΩ ± 0.12 kΩ	−0.0140
Sb:CuO	1300 kΩ ± 6.5 kΩ	325 kΩ ± 1.7 kΩ	90 kΩ ± 0.5 kΩ	40 kΩ ± 0.2 kΩ	−0.0630
Si:CuO	525 kΩ ± 2.7 kΩ	100 kΩ ± 5 kΩ	35 kΩ ± 0.18 kΩ	20 kΩ ± 0.1 kΩ	−0.0250

### 3.4. Gas Sensing Properties

**Figure 12:**
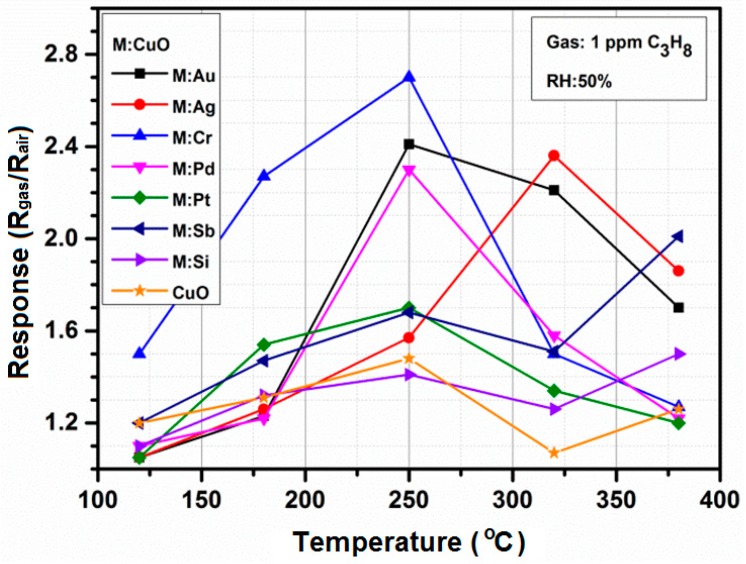
Gas response of the CuO- and M:CuO-based sensors (M: Ag, Au, Cr, Pd, Pt, Sb, Si) toward 1 ppm C_3_H_8_ (RH: 50%) as a function of working temperature ranging from 120 °C to 380 °C.

The dynamic sensing responses were measured using a homemade computer-controlled measurement system. The response (R) was defined as R_gas_/R_air_, where R_gas_ and R_air_ are the resistance of the sensor in propane and air, respectively. The response of CuO- and M:CuO-based (M = Au, Ag, Cr, Pd, Pt, Sb, Si) nanostructure sensors was measured toward 1 ppm C_3_H_8_ at 120 °C–380 °C (Figure 12). It is observed that the gas response of the sensors is greatly influenced by the working temperature due to the temperature-dependent gas adsorption and desorption on the oxide surface. The operating temperature was obtained by applying the power supply to a heater placed inside the sensor substrate. The formation of a uniform temperature distribution in the LTCC gas sensors was previously investigated and reported in [22]. The optimum working temperature was determined at 250 °C for all sensors, except the Ag:CuO-based one, which exhibits a maximum response at 320 °C. Further examining of the gas sensing characteristics has to be performed close to the optimum working temperature to find out the suitable working temperature. As is known, the operating temperature is an important parameter for gas sensors, because it determines the power dissipated by the heater necessary for the achievement of the optimal gas-sensing characteristics, and, through this parameter, influences the reliability and durability of solid-state gas sensors. For practical devices, one wishes to minimize the power needed to operate, so the lowest operating temperature is desired. The power consumption can be also reduced by optimizing the gas sensor substrate dimensions and geometry [22]. In an atmosphere containing flammable gases, a low temperature is favored also for safety reasons. Recently, researchers have investigated the possibility to fabricate sensors working at room temperature, e.g., [27,28]. However, such sensors exhibit low repeatability due to the lower desorption process. The dynamic sensing measurements of CuO- and M:CuO-based sensors were performed to evaluate their gas sensing properties in terms of sensitivity, response and recovery time(s), as well as to determine the reversibility for long-term measurements.

**Figure 13:**
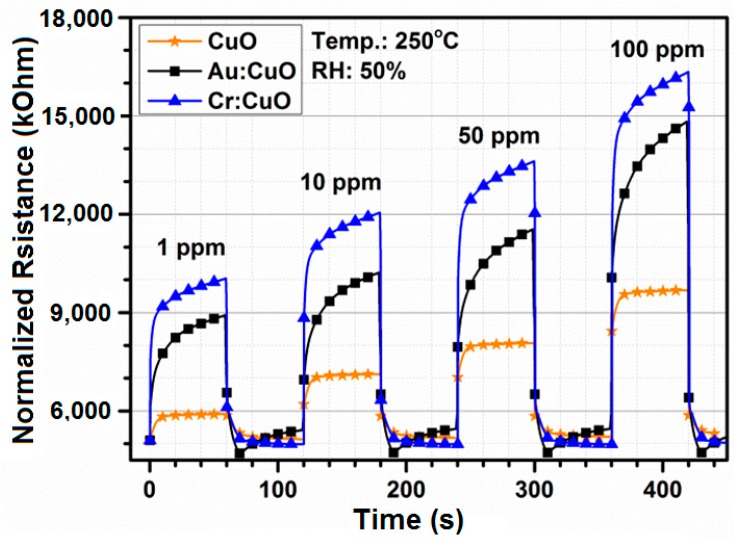
Propane curves of the CuO-based, Cr:CuO-based and Au:CuO-based sensor at 250 °C at multiple concentrations.

**Figure 14:**
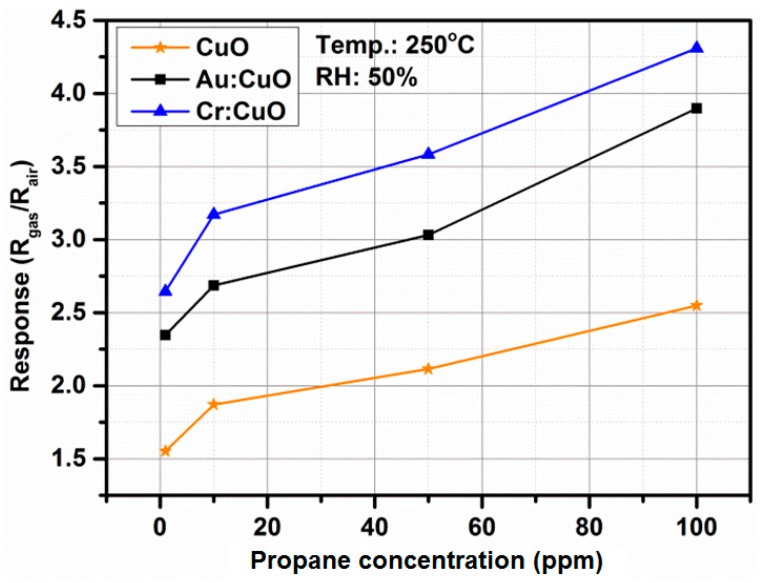
The gas response of the CuO-based, Cr:CuO-based and Au:CuO-based sensors as a function of propane concentration.

The propane-sensing curves show that the CuO- and M:CuO-based sensors exhibit a p-type response to propane. Figure 13 shows the responses (normalized resistance changes) for CuO-, Au:CuO- and Cr:CuO-based sensors (sensors with the highest responses upon exposure to C_3_H_8_) toward various propane concentrations measured at 250 °C and at relative humidity ~50%. The resistance of the measured sensors in air is low, which promptly increases and reaches a near plateau upon exposure to propane. The corresponding response of the measured sensors to propane as a function of concentration is shown in Figure 14. With increasing concentration of propane, the responses greatly increase. However, as was mentioned in the Introduction, for medical applications, the responses at the lowest concentration are the most valuable. The sensors have been previously stabilized at the working temperature for 24 h.

Kim *et al.* [29] investigated a selective detection of NO_2_ using Cr-doped CuO nanorods. The authors observed that pore volumes over the entire pore size range increased for a higher Cr concentration. This is beneficial for two reasons: first, for the surface area to volume ratio and, second, for the gas accessibility, which can explain the enhanced response of CuO nanostructures [29]. The Cr:CuO-based sensor exhibits higher resistance in air than the CuO-based sensor (Table 2) with the absence of the Cr_2_O_3_ peak (Figure 4), which suggests that Cr was incorporated into the CuO lattice. However, a detailed study of the sensing mechanism of Cr:CuO-based sensors would be necessary in order to determine the impact of various Cr concentrations. Based on the literature and the obtained results, the enhancement of responses to propane by Cr doping can be explained in part by the incorporation of Cr^3+^ into the CuO lattice and the consequent decrease in its hole concentration.

### 3.5. Response-Recovery Characteristics

**Figure 15:**
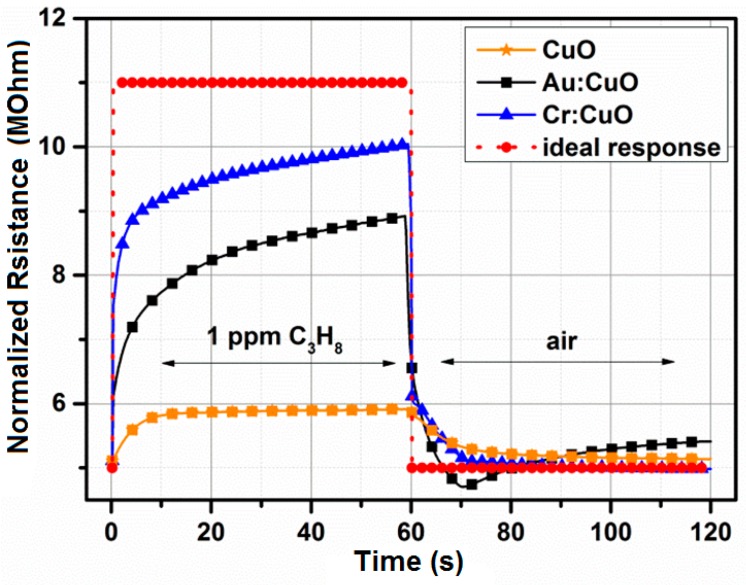
Response and recovery step of the resistance of the CuO-, Au:CuO and Cr:CuO-based sensors toward 1 ppm C_3_H_8_ at 50% RH and 250 °C.

Response and recovery times are one of the most important issues that have to be taken into account during the gas sensing measurements; therefore, they are well described in the literature, e.g., [30]. The response and recovery time(s) is the time to reach 90% variation of the sensor resistance upon exposure to an analyte gas and air, respectively. Usually, the times are determined by measuring changes in the electrical resistance from the base level in air (*R*_air_) to a steady level in air containing the analyte gas (*R*_gas_) upon switching the gas atmosphere from air to the analyte gas. However, the dead volume of a conventional chamber is too large to quickly introduce gases into the chamber within a sufficiently short time. The response and recovery time(s) have to be compared to the information of the chamber volume, the temperature, the thickness of the sensitive layer, *etc.* In this study, the authors used a quartz tube-shaped chamber having a volume approximately 40 cm^3^ ± 0.6 cm^3^. Savovic *et al.* [31] have reported the investigation results on gas diffusion dynamics of the response and recovery processes of a thin film semiconductor gas sensor using an equivalent model of a thin film device and a simple diffusion equation [31]. Park *et al.* [32] have reported that response and recovery times for the CuO nanocubes with 800 ppb formaldehyde, which were 50 s and 150 s at 250 °C, respectively. Liang *et al.* [33] have reported that the response time for the CuO-In_2_O_3_ nanofiber sensor toward 5 ppm H_2_S was 150 s at 250 °C. Kim *et al.* [34] have reported the response and recovery times for the bare CuO and Pd-functionalized CuO nanorods under exposure of H_2_S (20–100 ppm). The times were in the range of 80–700 s. Briefly, the recovery time of the Pd-functionalized nanorods sensor was 5–8-times shorter than that of the bare CuO nanorod sensor, while the response time was 2–3-times longer [34]. Abaker *et al.* [35] have reported that the response time for the CuO nanocubes sensor toward 5 × 10^−9^ mol·L^−1^ 4-nitrophenol was 10 s [35,36]. Based on a literature review, it is very difficult to directly compare the obtained results with the results presented by others without access to the actual raw experimental results and without considering the target application. Currently, the authors have focused on gas sensors for the portable breath analyzer where the response and recovery time(s) are as much important as sensitivity. Figure 15 shows the response and recovery step of the resistance of the CuO-based, Au:CuO-based and Cr:CuO-based sensors toward 1 ppm C_3_H_8_ at 250 °C (RH: 50%). Table 3 shows the response and recovery time(s) for 50 nm M:CuO-based nanosensors under exposure of 1 ppm propane.

**Table 3:** The response and recovery time (s) obtained for 50-nm films deposited at 100 °C and annealed at 500 °C for 4 h in air under exposure to 1 ppm C_3_H_8_ at the temperature with the maximum sensor response (see Figure 11).

Thin Film	C_3_H_8_ (1 ppm)	
	T_recovery_ (s)	T_response_ (s)
CuO	20	12
Au:CuO	30	34
Ag:CuO	26	18
Cr:CuO	10	24
Pd:CuO	35	15
Pt:CuO	55	12
Sb:CuO	92	30
Si:CuO	150	100

### 3.6. Long-Term Stability

**Figure 16:**
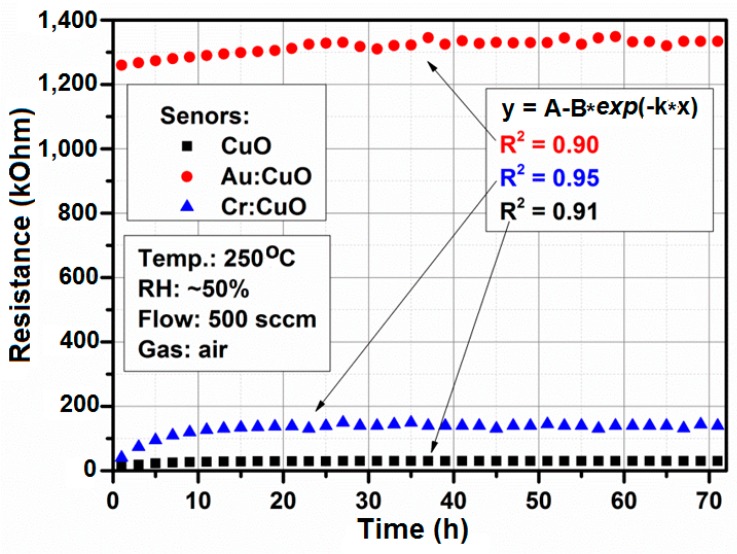
The baseline resistance change in time for the CuO-, Au:CuO- and Cr:CuO-based sensors for typical working conditions.

There are two adverse effects that may appear when the sensor works for a long period of time, *i.e.*, drift of the baseline signal (defined as the conductance in air or in a reference gas) and drift in the sensor response. The long-term stability measurements are very important for the practical use of a sensor. Therefore, it is necessary to perform many thermal treatments and cycle calibrations before fabrication. Figure 16 shows the baseline resistance variation of CuO-based, Au:CuO-based and Cr:CuO-based sensors for three days at 250 °C in ambient air. The measurement points were fitted by an exponential curve (*y = A − B ×* exp(*−kt*), where *A*, *B* and *k* are the experimental coefficients) with high values of the coefficient of determination *R*^2^: 0.90–0.95. It seems that such sensors will be stable enough to conduct several dozens of measurements over a long period of time. Commercially available gas sensors usually require a precondition period from 48 h up to seven days. During the long-term stability measurements, the working temperature was set to 250 °C; relative humidity was stabilized at 50%, and the air flow rate through the gas sensor chamber was set to 500 sccm. Previous experiment results with CuO-based and M:CuO-based thin films have shown good stability [23].

## 4. Conclusions

The investigation of metal oxide gas sensors was started over 40 years ago, and now, it seems to be constantly improving. However, the main research goals in MOX (Metal Oxide) gas sensors have been realized, such as improving the sensitivity, selectivity and stability for developed gas sensors. Moreover, the novel potential applications, e.g., in a portable breath analyzer, require extremely short response and recovery time(s) with very good selectivity and sensitivity. Therefore, with such a big variety of materials that can be used, the selection of optimal sensing material becomes a key problem in both the design and manufacturing of gas sensors with the required operation parameters. In this study, the CuO- and M:CuO-based sensors (M = Ag, Au, Cr, Pt, Pd, Sb, Si) were presented. The gas-sensing characteristics were discussed for films with the highest response toward 1 ppm of propane, which can be considered as one of the biomarkers of diabetes. The results suggest that the sensing properties of the cupric oxide films are improved by the addition of M-dopants (except Si), which act as catalysts in propane sensors. The Cr:CuO-based structure, annealed at 400 °C for 4 h in air, showed the highest sensor response, of the order of 2.7 at an operation temperature 250 °C. The response and recovery time(s) were: 10 s and 24 s, respectively. Furthermore, a detailed study of the sensing mechanism of the Cr:CuO-based sensor is needed, especially with an impact of various Cr concentrations. All measured sensors were obtained keeping the deposition parameters constant, due to the various dopant concentrations being used. The dopants are capable of improving the sensor properties by the formation and stabilization of smaller grains, by increasing the nanostructure porosity and by enhancement of the long-term stability.
